# Monitoring frequency of intra‐fraction patient motion using the ExacTrac system for LINAC‐based SRS treatments

**DOI:** 10.1002/acm2.12279

**Published:** 2018-03-25

**Authors:** Benjamin C. Lewis, William J. Snyder, Siyong Kim, Taeho Kim

**Affiliations:** ^1^ Department of Radiation Oncology Virginia Commonwealth University Richmond VA USA

**Keywords:** brainlab ExacTrac, intra‐fraction imaging, SRS, stereotactic radiosurgery

## Abstract

**Purpose:**

The aim of this study was to investigate the intra‐fractional patient motion using the ExacTrac system in LINAC‐based stereotactic radiosurgery (SRS).

**Method:**

A retrospective analysis of 104 SRS patients with kilovoltage image‐guided setup (Brainlab ExacTrac) data was performed. Each patient was imaged pre‐treatment, and at two time points during treatment (1st and 2nd mid‐treatment), and bony anatomy of the skull was used to establish setup error at each time point. The datasets included the translational and rotational setup error, as well as the time period between image acquisitions. After each image acquisition, the patient was repositioned using the calculated shift to correct the setup error. Only translational errors were corrected due to the absence of a 6D treatment table. Setup time and directional shift values were analyzed to determine correlation between shift magnitudes as well as time between acquisitions.

**Results:**

The average magnitude translation was 0.64 ± 0.59 mm, 0.79 ± 0.45 mm, and 0.65 ± 0.35 mm for the pre‐treatment, 1st mid‐treatment, and 2nd mid‐treatment imaging time points. The average time from pre‐treatment image acquisition to 1st mid‐treatment image acquisition was 7.98 ± 0.45 min, from 1st to 2nd mid‐treatment image was 4.87 ± 1.96 min. The greatest translation was 3.64 mm, occurring in the pre‐treatment image. No patient had a 1st or 2nd mid‐treatment image with greater than 2 mm magnitude shifts.

**Conclusion:**

There was no correlation between patient motion over time, in direction or magnitude, and duration of treatment. The imaging frequency could be reduced to decrease imaging dose and treatment time without significant changes in patient position.

## 1 INTRODUCTION

Frameless stereotactic radiosurgery (SRS) has taken on a significant role in treatment of cranial lesions, including primary and metastatic brain tumors, nerve disorders, and arteriovenous malformations. SRS provides an alternative to surgery, and whole brain radiotherapy (WBRT), or can accompany these treatments to ensure residual tumor cells are eliminated. Due to the high dose, sharp dose gradients, and small margins utilized in SRS, accurate patient positioning is vital to reduction in dose to normal tissue, as well as tumor control.^1^ To achieve the required levels of setup accuracy, image guidance and a thermoplastic mask attached to the treatment couch are used in place of an invasive head frame. Previous works have shown that intra‐fractional positioning accuracy of mask‐based immobilization systems range from 1.59 ± 0.84 mm to 4.7 ± 1.7 mm using a thermoplastic mask and image guidance from Cone‐beam CT (CBCT), CT simulation, portal images, and biplanar diagnostic x ray.^2^, ^3^, ^4^, ^5^ These positioning errors are still too large for SRS treatments, due to irradiating critical organs during the treatment. A study by Kim et al. measured the intrafraction shift of 16 patients and found the average to be 0.39 mm, however, this small shift resulted in an average variation in maximum dose to organs at risk (OAR) of 7.15%.^6^ Image guidance significantly reduces the setup errors, and is essential for accurate delivery of SRS. Multiple systems have been developed for image guidance, including electronic portal imaging devices (EPIDs), stereoscopic kV imaging, CBCT, and MVCT.^7^ A study by Ramakrishna et al. investigating intra‐fractional motion found that there was less than 1.0 mm discrepancy between frame‐based and image‐guided at initial setup using a stereoscopic kilovoltage x ray system combined with an infrared position tracking system, and a positioning error of 0.7 mm for image‐guided setup.^8^ These imaging methods are highly reliant on bony anatomy for alignment due to the inability to distinguish brain metastases. Previous works have determined that the skull is a reliable surrogate for tumor position.^2^, ^9^, ^10^

This study investigates the intra‐fractional motion during SRS treatment utilizing a thermoplastic mask and repositioning during treatment using ExacTrac stereoscopic kV x ray system based on our institutional imaging protocol.

## 2 METHODS

**Figure 1:**
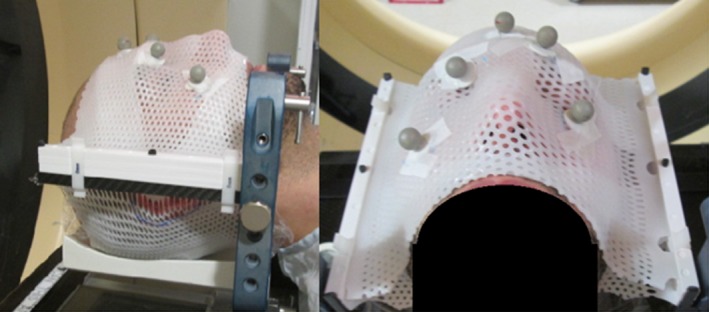
An image of a thermoplastic mask used in our clinic for SRS procedures, showing all five IR markers.

**Figure 2:**
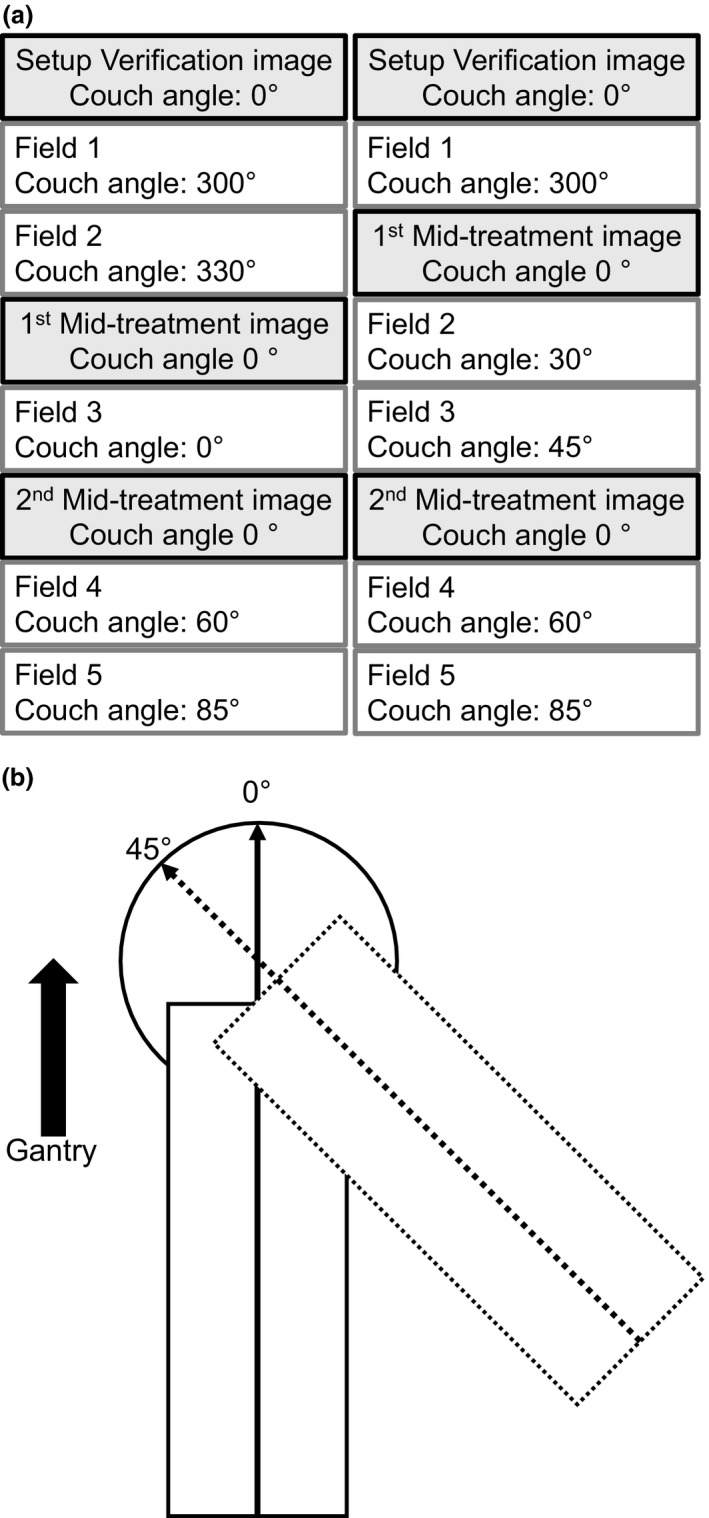
SRS treatment design. (a) SRS treatment field delivery, image acquisition, and couch rotation for two treatment examples. The 2nd mid‐treatment image in the left treatment example was captured at a couch angle of 60° if the IR markers were not obstructed by the gantry, if the markers were obstructed, then the couch was rotated to 0° for image acquisition. (b) the IEC scale used for couch rotations in this study. The solid rectangle and line with arrow head indicate couch position at couch angle 0°. The dotted rectangle and arrow indicate couch position at couch angle 45°.

A total of 104 sequential patients who had undergone single fraction SRS treatment for brain tumors were retrospectively chosen for this study. All patients had been treated on a clinical Linear accelerator (Trilogy, Varian Medical Systems, Palo Alto, CA), with a thermoplastic mask used for patient immobilization, and image guidance using the ExacTrac kV X‐ray system and ExacTrac software version 5.5 (Brainlab, Munich, Germany). Thermoplastic masks are from BrainLab, model 41100, and cover from the patient's forehead, to just above the upper lip. No bite block is used for mask positioning. Thermoplastic masks were formed after heating in a water bath, immediately prior to CT‐simulation, an image of an example mask is shown in Fig. 1 All patients were imaged three times over the course of treatment, once pre‐treatment, and twice during treatment (1st and 2nd mid‐treatment). Patients were initially setup using the in‐room lasers and infrared markers, then the pre‐treatment image was acquired. After the pre‐treatment image, if any shift was required a second x ray was acquired for shift verification. For mid‐treatment images, a verification x ray was acquired for shifts > 2.0 mm in magnitude. Mid‐treatment images occurred between treatment fields and couch rotations, in one of two configurations shown in Fig. 2(a). Figure 2(b) displays the International Electrotechnical Commission (IEC) scale for the treatment linear accelerators used in this study.

After each image acquisition, the 3D shift was calculated by the ExacTrac system to correct setup error relative to planning digitally reconstructed radiographs (DRRs) and applied. The longitudinal, lateral, and vertical shifts, were retained for every image. Rotational corrections were not applied because a 6D couch was not in use. Time between image acquisition and translational shift values were analyzed to determine trends in shift magnitude, and correlation with time between acquisitions. The sample Pearson correlation coefficient (*r*) was used to determine if any linear correlation between variables existed, which can be interpreted as values equal to 1 indicate that a linear equation perfectly describes the relationship between the two data sets, equal to −1 indicate that a negative slope linear equation describes the relationship, and values near zero indicate no linear correlation.

## 3 RESULTS

**Figure 3:**
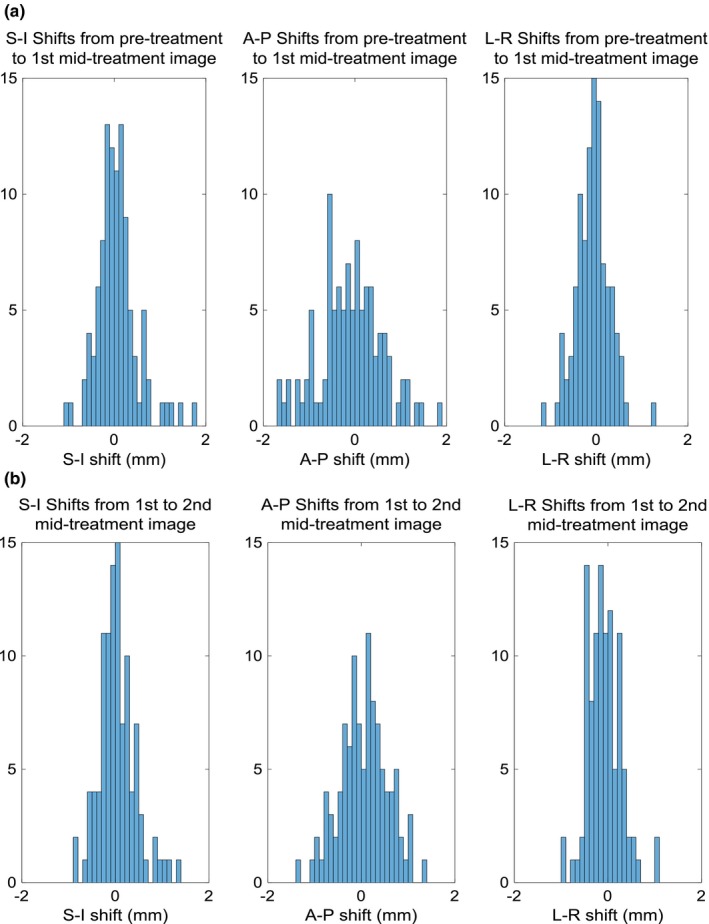
Directional shift histograms. Directional shifts from (a) pre‐treatment image to 1st mid treatment image sets, and (b) 1st to 2nd mid‐treatment image sets.

**Figure 4:**
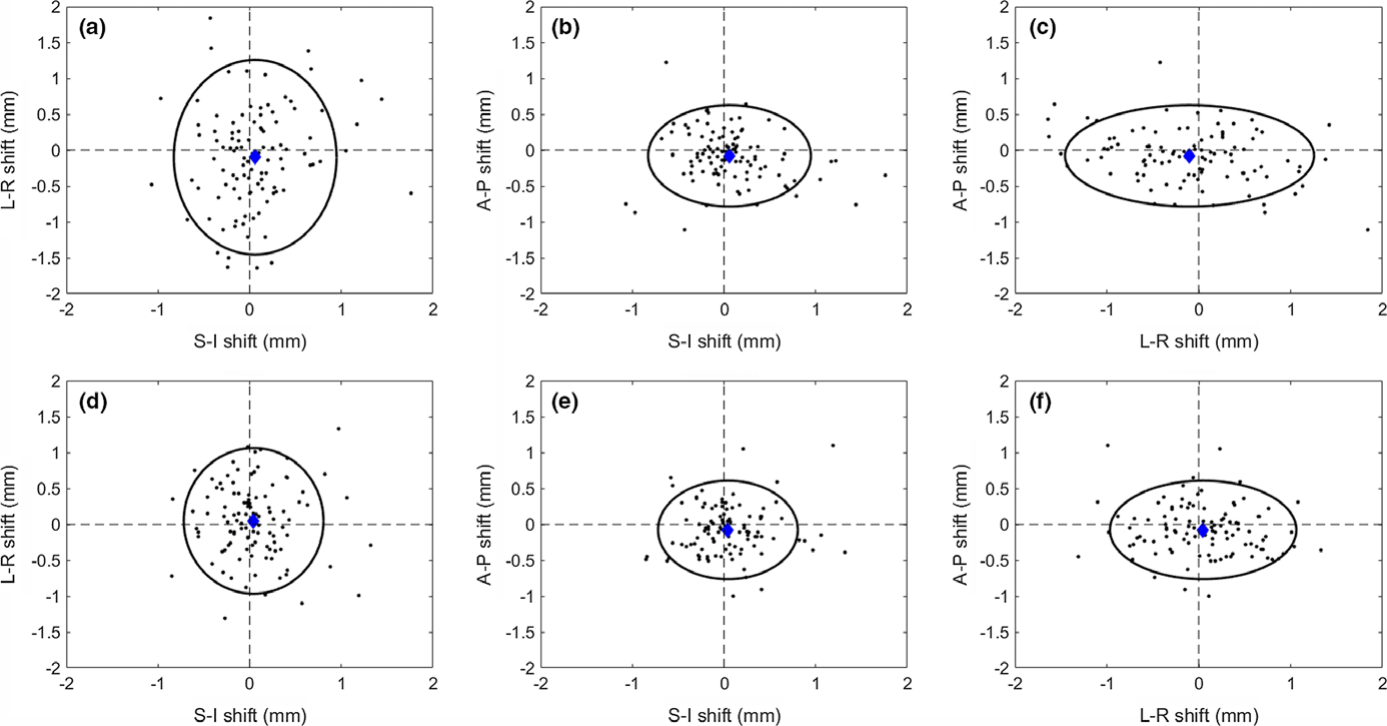
Shift 3D distribution. Calculated shifts for all 104 patients from the 1st mid‐treatment image (a‐c), and 2nd mid‐treatment image (d‐f). The mean (blue diamond), and 95% confidence region (black ellipse) are indicated.

The average shifts for all directions were less than or equal to 0.15 mm over all imaging time points, however, the magnitude translations were 0.64 ± 0.59 mm, 0.79 ± 0.45 mm, and 0.65 ± 0.35 mm for pre‐treatment, 1st mid‐treatment, and 2nd mid‐treatment image, respectively, as shown in Table 1. Table 2 shows the average translations in the superior–inferior (S‐I), anterior–posterior (A‐P), and left–right (L‐R) directions for each image acquisition. The small average value and large standard deviation of patient shifts suggests that the direction of the patient motion between imaging points is approximately randomly distributed about the initial position, and is limited by the strain of the thermoplastic mask on either side, shown in Fig. 3. Figure 4 plots all imaging points, average value, and 95% confidence region for 1st and 2nd mid‐treatment image shifts.

**Table 1:** Patient shifts calculated by the ExacTrac system for each imaging point during treatment. These values indicate the directional shifts from the ExacTrac system

	S‐I shift (mm)	A‐P shift (mm)	L‐R shift (mm)	Magnitude (mm)
Pre‐treatment	−0.02 ± 0.34	−0.05 ± 0.68	−0.06 ± 0.42	0.64 ± 0.59
1st mid treatment image	0.06 ± 0.45	−0.10 ± 0.69	−0.08 ± 0.36	0.79 ± 0.45
2nd mid treatment image	0.04 ± 0.39	0.05 ± 0.52	−0.08 ± 0.35	0.65 ± 0.35

**Table 2:** Patient translations calculated by the ExacTrac system for each imaging point during treatment. These values indicate the magnitude translations from the ExacTrac system

	S‐I shift (mm)	A‐P shift (mm)	L‐R shift (mm)	Magnitude (mm)
Pre‐treatment	0.23 ± 0.25	0.26 ± 0.33	0.42 ± 0.54	0.64 ± 0.59
1st mid treatment image	0.33 ± 0.32	0.28 ± 0.24	0.55 ± 0.43	0.79 ± 0.45
2nd mid treatment image	0.28 ± 0.27	0.28 ± 0.22	0.42 ± 0.31	0.65 ± 0.35

**Figure 5:**
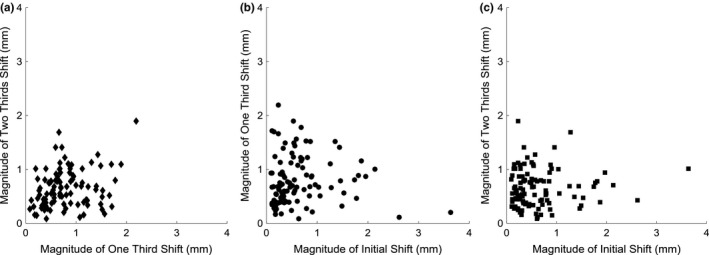
Magnitude shifts at varying time points. Magnitude shifts for each patient, comparing the magnitude between imaging time points. (a) Plots the magnitude shifts for 1st and 2nd mid‐treatment image acquisitions. (b) plots the magnitude shifts for pre‐treatment and 1st mid‐treatment image acquisitions. (c) plots the magnitude shifts for pre‐treatment and 2nd mid‐treatment image acquisitions.

Figure 5 shows the magnitude shifts plotted against other imaging points. There is little to no correlation between the points, indicating that a magnitude shift at one time point does not result in a shift of similar magnitude at another time point. *r* values are 0.2470, −0.0235, and 0.0534 for 1st and 2nd mid‐treatment images, pre‐treatment and 1st mid‐treatment images, and pre‐treatment and 2nd mid‐treatment images respectively. Of the 104 2nd mid‐treatment images acquired, 20 had shifts greater than 1 mm in magnitude, 2 greater than 1.5 mm, and none greater than 2 mm in magnitude.

**Figure 6:**
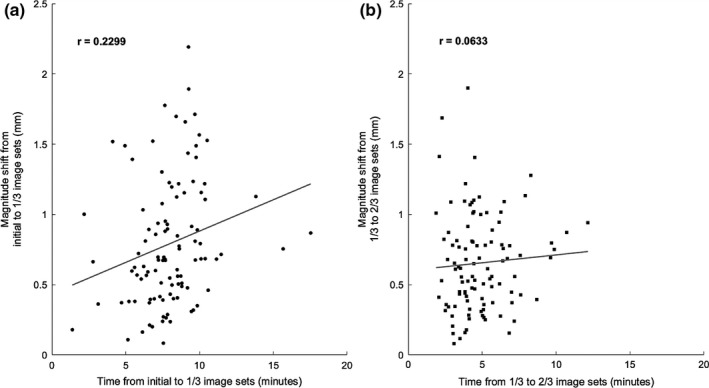
Magnitude shifts over time. The magnitude shift against time between image acquisitions for (a) pre‐treatment to 1st mid‐treatment image acquisition, and (b) 1st to 2nd mid‐treatment image acquisition.

Figure 6 compares magnitude shift to length of time between image acquisitions for shift from the initial to 1st mid‐treatment image, and 1st mid‐treatment image to 2nd mid‐treatment image for Figs. 6(a) and 6(b), respectively. The *r* values are 0.2299, and 0.0633 for initial to 1st mid‐treatment image, and 1st mid‐treatment image to 2nd mid‐treatment image respectively.

## 4 DISCUSSION

Multiple methods have been developed to immobilize, and accurately align the target volume with the radiation treatment isocenter. Due to the possibility of patient motion with non‐invasive frameless immobilization systems, imaging techniques must be applied throughout treatment to ensure that dose is delivered and distributed according to the treatment plan. Previous works have found that image‐guided frameless SRS provides a similar level of intrafraction patient motion as frame based SRS, but that a single image acquired pre‐treatment is not sufficient to monitor patient motion.^3^, ^8^, ^11^ This study found average magnitude shifts of less than 0.8 mm for all image acquisitions, with a range of 0.08–3.64 mm, similar to previous works. However, it is important to note that values for averaging of magnitude translations, and averages of negative and positive shifts give significantly different values, and errors in separate parts of setup error. The average of shifts gives insight into possible systematic errors within the treatment process, while the average of absolute translations allows for analysis of patient motion during treatment. An average shift very close to zero, and a symmetric distribution about the isocenter, indicate that the setup errors had a very small systematic component.

This study had the limitation of using ExacTrac software version 5.5, which requires all IR markers to be visible for image registrations. For this reason, x ray acquisitions could only occur at specific couch and gantry angles. Couch motion around isocenter was checked during monthly QA of the LINAC, and walkout was found to be negligible for angles < ±30°, and clinical protocol called for all images to take place within this range. In addition to these measurements, the ExacTrac system was calibrated on a monthly basis, and a Winston‐Lutz test was performed daily for verification. Another limitation, was the limited number of image acquisitions with greater than 10 min between images. The data should not be extrapolated past this time point, however, with fewer image acquisitions a single treatment would most likely take less than 20 min to complete in the experience of our institution.

When magnitude translation was compared to time between image acquisitions, no statistically significant trend was found. The maximum magnitude translation was 3.64 mm, seen in Fig. 5, and the maximum single direction shift was 3.62 mm in the L‐R direction, which is not depicted in the plots because it occurred during the initial setup. These maximum values occurred during the pre‐treatment image, and correspond to the same patient. The large L‐R variations seen in this study may have been a result of weight loss from the time of CT‐simulation to treatment, however, this was not investigated by this work. Based on the Pearson correlation coefficients, the null hypothesis that the variables were not correlated could not be rejected with a confidence level of *P* = 0.05 for the comparison of magnitude shift between pre‐treatment and 1st mid‐treatment images, and pre‐treatment and 2nd mid‐treatment images, or for the comparison of magnitude shift to length of time between image acquisitions for the 1st mid‐treatment image to 2nd mid‐treatment image. The null hypothesis could not be rejected with a confidence level of *P* = 0.02 for comparison of magnitude shift between 1st and 2nd mid‐treatment images, or for the comparison of magnitude shift to length of time between image acquisitions for initial to 1st mid‐treatment images. Because there is no significant correlation between imaging frequency, and magnitude translation, the magnitude of motion is bounded by the mask, within the clinical tolerance.

## 5 CONCLUSION

This study investigates intra‐fractional motion in SRS, evaluating the necessity and frequency of monitoring intra‐fractional setup changes in frameless SRS patients. Although the dosimetric impact of these imaging sets is low, the process adds to the overall patient time on table, introduces the potential for error, and it is always advantageous to reduce imaging dose to the patient. Reducing imaging frequency would reduce the required treatment time, which is a concern due to the use of a thermoplastic mask covering the patient's face during treatment that can cause nervousness or discomfort. At our institution with our current practice, it is reasonable to reduce imaging frequency to one pre‐treatment image, and one mid‐treatment image, occurring approximately halfway through treatment delivery.

## CONFLICT OF INTEREST

No author has any conflicts of interest.
